# On Removal of the Entire Tongue

**Published:** 1869-03

**Authors:** Sampson Gamgee

**Affiliations:** Surgeon to the Queen's Hospital, Birmingham.


### ARTICLE IX.

### *On Removal of the Entire Tongue.*

By Sampson Gamgee, F.R.S. (Edin.), Surgeon to the Queen's Hospital, Birmingham.

Little more than twenty years have elapsed since a sur geon so bold and skilful as Mr. Liston spoke and wrote of ovariotomists as "belly-rippers," who deserves to be criminally indicted whenever they again attempted the operation of ovarian extraction ; which he regarded as a hopeless, nay barbarous, procedure. On the experience of several hundred cases, ovariotomy is now established as one of the most legitimate operations of surgery.

Removal of the entire tongue for cancer has been frequently attempted, and several times accomplished; but the result has been so generally unfortunate, that Mr. Gross can scarcely be said to have exaggerated the verdict of judicious surgeons when he concluded a brief survey of methods for the removal of the tongue with these words:—"I have certainly not alluded to this operation with a view of recommending its adoption; on the contrary, it cannot be too strongly condemned. If there is any operation in surgery that deserves to be stigmatised as cruel and unnecessary, it is this."

With this opinion before me, supported as it was by such experience as Mr. Syme's, I did not, as I do not still, accept as conclusive the evidence on which the great majority of surgeons condemn removal of the entire tongue as an unjustifiable operation; I deem their reasons insufficient, since the researches on which they are based, pathologically and bibliographically, are scanty, superficial, and inadequate. As a rule, matters are little studied which do not offer a prospect of speedy profitable return. When ovariotomists were, in the graphic language of Mr. Liston, styled "belly-rippers," the anatomy, diagnosis, and clinical history of ova rian disease were little studied: for the prospect had no allurements

Without futher introduction, I shall condense from the copious notes of our resident surgeon, my friend Dr. Robert Jolly, the history of a case in which I lately removed the tongue— with no better result, I hasten to avow, than most of my predecessors; but I hope the event may prove with this difference: that the case will become the starting-point and basis of an inquiry which may, at no distant date, result in a definite settlement, after comprehensive inquiry, of the merits and demerits of the different methods of removing the tongue, wholly or in part.

*History of case.*—Henry S.——, aged fifty-two, a laborer, was admitted into the Queen's Hospital, from Stratford-on-Avon, on the 25th of August last. The patient, a spare-built but florid-looking man, was married and the father of seven living children. He had always enjoyed excellent health up to twelve months last past, when he began to experience shooting pains in the ear and down the neck Shortly afterwards his tongue became sore, and on examining it he found a small, well-defined, roundish lump with ulcerated surface, on the left side of the tongue. It has continued to extend ever since, and has occasioned great pain and difficulty in swallowing. About three month after its first appearance he applied to a medical man, who extracted two sound teeth in the lower jaw because they hurt the tongue. Five months later (four months before admission) the disease began to spread with considerable rapidity, and bled very much. When first seen by me, the patient had a deep excavated ulcer, of oval shape, involving the anterior two thirds of the left side of the tongue. The edges of the ulcer were thick, raised everted, and irregularly notched; its floor was foul, and studded with pea-shaped, tuberous granulations, discharging a thin, ichorous fluid. There was great fetor of breath, and profuse salivation. No implication of structures at the floor of the mouth, or apparently of neighboring glands. General health much impaired, in consequence of inability to take solid food.

A great variety of means were employed with a view to improve the general health and character of the ulcer, but as they all failed, I operated on the 3rd of October. The patient having been previously shaved, was placed upon a table, and rendered completely insensible with chloroform administered by Mr. Snow and Dr. James Hinds. The shoulders being raised and the head well thrown back, standing on the right side of the patient I made a semilunar incision along the base of the lower jaw, commencing at the symphysis, and extending it outwards on either side to a point just anterior to the facial artery. A second incision was carried vertically downwards from the centre of the jaw to the hyoid bone, at right angles to the first. The triangular flaps thus marked out (consisting of skin, areolar tissue, and the anterior fibres of the platysma myoides) were directed down. A narrow-bladed knife was then thrust in the mesian line close behind the bone, from below into the mouth, and swept along the inner surface of the lower jaw, as far as the posterior limits of the first incision, to divide the attachments of the muscles and the buccal membrane. An opening of sufficient extent being thus effected into the floor of the mouth, the tongue was drawn down upon the anterior part of the neck, and secured by my friend and colleague, Mr. J. F. West. The tongue being raised, I thrust a narrow bladed knife through the raphé from below, just in front of the hyoid bone, into the mouth. Withdrawing the blade, I passed one by one two ecraseurs through the wound, fixing one on the right half of the tongue, the other on the left, just in front of the anterior pillars of the fauces; the left one was tightened clearly, though only a couple of lines, behind the posterior limits of the disease. My colleagues, Mr. Furneaux Jordon and Mr. J. St. S. Wilders, took respective charge of the left and right ecraseurs, tightening each alternately, at intervals varying from half a minute to a minute. Though the process of division was slow, no sooner did the ecraseurs become detached than a large quantity of blood welled out of the wound, and the patient became very pale. I introduced my thumbs into the fauces bringing forward the mucus membrane and grasping it together with the flaps which had been dissected down from the submental region. I thus attained two objects: compressing the bleeding surfaces and bringing the tongue well forward; the flapping epiglottis was in full view, and the risk of blood entering the trachea was averted. The surface of the wound and the stump of the tongue were immediately mopped with styptic colloid, a triangular lump of ice held in the mouth with a pair of forceps, long hot flannel stockings slipped on the arms, a hot blanket wrapped round the legs, and a brandy injection at once administered per rectum. After all oozing had ceased, the patient was carried from the theatre to the ward, where another brandy and beeftea injection was given at once.—3 P.M. (three hours after operation): Reaction fully established; skin hot and moist; pulse 120, full; temperature $102\frac{1}{5}°$ respiration 36. Injection of beef-tea and brandy to be continued every two hours.—7 P. M.: Pulse 134; temperature 104°; respiration 28. The glazed flaps were gently brought together and made to meet by silver sutures in the transverse line; the vertical incisions were likewise approximated above, the lower angle being left open for free escape of secretions.

Oct. 4th.—9 A. M.: Passed a comfortable night; slept at intervals; no bleeding. Pulse 104; temperature $100\frac{3}{5}°$; respiration 32. Injections to be continued every fourth hour. —10 P.M. Hæmorrhage rapid and profuse from the mouth. The quantity lost must have been considerable, for not only was the pillow saturated with blood, but a good deal was spilt on the floor. Dr. Jolly staunched the flow by steady pressure on both carotids for about two minutes, which produced instant syncope. A short time after a warm water and brandy injection the circulation became developed, and there was no recurrence of the bleeding.

5th.—Free from pain; wound looks well, and is for the most part healed. Thirst relieved by moistening his lips with ice: drank a cup of milk this morning. Pulse 110; temperature $101\frac{2}{5}°$; respiration 28.

6th.—Had an opiate last evening; nevertheless the night has been somewhat restless. Pulse 124; temperature $102\frac{2}{5}°$; repiration 24. Injections discontinued. The patient takes a cupful and a half of milk every four hours. To correct fetor of breath the following gargle was ordered (and to be kept iced): Chlorate of potash, one drachm and a half; borax and honey, one ounce; rose water, two ounces; to eight ounces of water.

7th.—Wound looks perfectly healed, except at lower angle of vertical incision, where the secretions from the mouth escape. Pulse 118, temperature $100\frac{4}{5}°$; respiration 32. Altogether the patient is going on most favorably. To have two or three eggs daily, to be beaten up with milk which is taken freely.

8th.—Was frequently disturbed during the night by cough which was relieved by mustard plasters to the chest At times the man wanders, but speaks collectedly when aroused. Pulse 116; temperature $100\frac{4}{5}°$; respiration 28. Takes milk, arrowroot, and calf's-foot jelly freely.

9th.—Passed a restless night. Face flushed; expression of countenance anxious. Wound has opened up again along the base of the jaw, stitches removed and strapping applied. There is a considerable swelling below and behind the angle of the jaw on the left side. Pulse 128; temperature $101\frac{4}{5}°$; respiration 24.

10th.—Was restless during the night, and the stomach became exceedingly irritable, even to frequent vomiting. Ordered a draught, containing two drops of hydrocyanic acid, in a little mint water, which remained on the stomach; in an hour afterwards also to take some warm brandy-and water. Feels very low. Pulse 128, soft and feeble; temperature 101°; respiration 30.

11th.—Worse: debility increasing: features sunken: pulse 138, scarcely perceptible. Temperature $100\frac{3}{5}°$; respiration 37,

12th.—The patient gradually sank and died at 2 A.M this morning.

*Examination of the tongue.*—As I had the pleasure of seeing Mr. T. H Bartleet at the operation, I directed the tongue to be sent to him for examination. From the note with which he subsequently favored me I take these facts: that, on looking down on to the dorsum of the tongue, the right side appeared perfectly healthy while the left side presented a foul excavated sore, reaching posteriorly to within one-eighth of an inch of the line of incision, on the under surface to within two lines of the raphé, on the upper surface to within three lines of the middle line. The disease extends nearly to the tip. The left side of the tongue is greatly thickened, being an inch and a half in depth, while the healthy side is barely three quarters of an inch. Nearly the whole surface of the left of the tongue consists of an ulcer, with the edges sinuous and undermined, foul and sloughy. The diseased part was horny on section. On cutting horizontally through the tongue, tubercular nodules were seen extending from the deeper parts of the ulcer towards the median line, and apparently resting in healthy tissue. These nodules were firm, and of a yellowish-grey color. On microscopic examination of the juice obtained from a surface of the section, it was found to contain nucleated cells of the size of blood-cells, and free nuclei; also large epithelial cells of various shapes, with two or three nuclei each, some of the cells being spherical, others ovoid, fusiform, or polygonal. A thin section showed irregular nucleated cells, lying side by side, and held together by fine fibres. Some of the cells contained oil globules, and appeared shrunken. There were no distinct broad cells filled with large nuclei, and no laminated corpuscles.

*Autopsy*, Oct. 15th.—Stump of tongue in a healthy state; but the soft parts forming the floor of the mouth were in a very sloughy condition. Corresponding to the swelling behind and under left angle of jaw was an indurated lymphatic gland, a section of which presented characteristic cancer cells. Thoracic and abdominal organs perfectly healthy. Lungs crepitant throughout, sections from both bases floating well on water.

The rapid enlargement of the gland beneath the jaw after the removal of the tongue was a striking fact, though not noticed during life, the gland must have been slowly on the increase some time; and the malignant deposit with which it was infiltrated somewhat lessens regret at the poor man's speedy death, for he could not have survived the glandular disease many months. Such a complication militates against the operation, but, even if suspected, would not necessarily be a bar to it. Most surgeons have amputated limbs and removed other parts where the neighboring lymphatic glands have been enlarged, but they have regained normal dimensions on the substraction of the irritating cause. We can never be certain that such will be the case but the risk must often be taken, in the absence of special indications to the contrary.

With the incisions for the submental aperture according to Regnoli's directions I am quite satisfied, for they fulfil the great indication of giving plenty of room without involving considerable vessels or important structures; they permit of the safe administration of chloroform throughout the operation, bring into view the whole extent of disease, and place under command the possible sources of hæmorrhage. Prof. Regnoli ligatured the stump of the tongue in several portions before he excised, and his case made an excellent recovery; it was not until four years later that cancer returned in the tonsils and proved fatal. At the foot of my copy of Regnoli's pamphlet I find this note, made in Florence the 15th of April, 1852: "The author told me the day before yesterday that he has performed the operation six times; only one of the patients, an old woman, died after a few days. He does not know what became of the other four cases that survived."

With the écraseur I was not satisfied, and should not employ it again in a similar case. The bruising it inflicts is an evil only tolerable if the use of the instrument guarantees safety from hæmorrhage. The patient lost a good deal of blood during the operation, and would have died from secondary hæmorrhage the next day but for Dr. Jolly's prompt compression of the carotids. The écraseurs were certainly not tightened quite so slowly as M. Chassaignac directs; but Foacher has recorded a case* of partial removal of the tongue by two ecraseurs worked at intervals of one minute, so that fifty minutes were required to effect the section; nevertheless, hæmorrhage was so profuse as to necessitate ligature of the external carotid.

The merit of the operative method followed in this case,

---

*Quoted in the LANCET, 1863, vol. i., p. 79.

and of that which Professor Syme, Mr. Fiddes, and Dr. George Buchanan† have practiced, after the examples of Maisonneuve,‡ Huguier,§ Sédillot,‖ and Rizzoli,¶ is a question for experimental solution. These surgeons have all divided the soft parts in the middle line through the lip to the hyoid bone, commanding the tongue by sawing through the symphysis of the jaw. In order not to interfere with voluntary deglutition, Mr. Syme divided the hyoglossi, but kept intact the genio-hyoidei and the mylo-hyoidei—an economical provision very deserving of notice.

Notwithstanding Mr. Nunneley's successful case** of removal of the entire tongue by ligature passed from the under chin, and his advocacy of the écraseur introduced through a submental opening—a suggestion to which the cases of Arnott, Mirault, and others give countenance,—I incline to the opinion that, if the entire tongue is to be excised at all, it must be brought into full view, so as to command efficiently the limits of the disease and the sources of hæmorrhage. Cancer of the tongue is a disease so painful and fatal, the application of caustics and partial excisions offer so little promise, that the radical procedure merits further consideration with a view to judicial settlement of its propriety and plan. No cases remind one more than these do of the wise saying of the old Paris academicians: "L'opération n'est qu'un point dans l'exercise de la chirurgie." No cases stand in need of more precise pathological investigation, wider bibliographical research, and closer statistical scrutiny.—*Lancet.*

---

† The Lancet, 1860 to 1866.

‡ Gazette des Hopitaux, 1862, p. 568.

§ Abeille Medicale, N. 31, Novembre 5, 1851.

‖ Traite de Medicine Operatoire, tome second, p. 35. Paris, 1855.

¶ Procesio per la Demolizione della Lingua. Memorria del Professore Francesco Rizzoli. Bologna: Tipidi S. Tommaso d' Aquino, 1854.

** The Lancet, vol. ii, 1861.

